# Isolation of mycobacteria from clinical samples collected in the United States from 2004 to 2011

**DOI:** 10.1186/1746-6148-9-100

**Published:** 2013-05-08

**Authors:** Tyler C Thacker, Suelee Robbe-Austerman, Beth Harris, Mitchell Van Palmer, Wade Ray Waters

**Affiliations:** 1 United States Department of Agriculture, Infectious Bacterial Disease Research Unit, National Animal Disease Center, Agricultural Research Service, Ames, IA, USA; 2 United States Department of Agriculture, Mycobacteria and Brucella Section, National Veterinary Services Laboratories, Ames, IA, USA

## Abstract

**Background:**

Mycobacteria other than *M. bovis* may interfere with current bovine tuberculosis diagnostic tests resulting in false positive test results. As the prevalence of *M. bovis* decreases in the United States, interference from other mycobacteria play an increasingly important role in preventing the eradication of *M. bovis*. To identify mycobacteria other than *M. bovis* that may be interfering with current diagnostic tests, a retrospective study was performed to identify mycobacteria isolated from clinical tissues at the National Veterinary Services Laboratories between 1 January 2004 and 9 October 2011.

**Results:**

During the study period, 2,366 mycobacteria other than *M. bovis* were isolated from samples submitted for clinical diagnosis of *M. bovis*. Fifty-five mycobacterial species were isolated during this time period. In cattle, *M. avium* complex, *M. fortuitum/fortuitum* complex, *M. smegmatis, M. kansasii,* and *M. terrae* complex were the predominate species other than *M. bovis* isolated from tissues submitted for culture. Mycobacteria other than *M. bovis* isolated from deer were predominantly *M. avium* complex, *M. terrae/terrae* complex, and *M. fortuitum/fortuitum* complex.

**Conclusions:**

These data provide information characterizing the species and relative prevalence of mycobacteria other than *M. bovis* that may interfere with current diagnostic tests.

## Background

Infection with *Mycobacterium bovis* (*M. bovis*) can result in tuberculosis in many mammalian species including humans. The risk of zoonotic transmission of *M. bovis* from animals to humans has led to the development of the Bovine Tuberculosis eradication program in the United States [1,2]. Discovery of *M. bovis* infected livestock and/or captive wildlife is reportable in the United States and results in quarantine of animals and animal products costing livestock producers and United States Government millions of dollars annually [2,3].

Antemortem diagnosis of tuberculosis in animals is based on immunological responses (skin test or *in vitro* cell based assays) to a purified protein derivative (PPD). PPD derived from *M. bovis* (PPDb) is produced by precipitating protein from heat-killed cultures of a laboratory-adapted strain of *M. bovis*, AN5. PPD’s are a complex mixture of proteins, carbohydrates, and lipids [4] that share homology with other mycobacteria and even other bacteria ([5-8]). A number of techniques have been used to evaluate AN5 and other strains of *M. bovis* including *M. bovis* BCG to discover specific antigens to replace the complex mixture found in PPD. Several antigens have shown considerable promise in existing and emerging diagnostic assays (e.g. MPB70, MPB83, ESAT6, CFP10, and OmpTb) [9-12]. However, cross-reactivity with other mycobacterial species [13,14] and variation in the immune response from animal to animal [15,16] continues to hamper improved diagnostic specificity [3].

A key component to development of improved diagnostic assays is to identify proteins or nucleic acid sequences that are specific to *M. bovis*. Ideally these are only produced by *M. bovis* and are significantly different from those produced by other mycobacteria. To date most diagnostic targets have been identified using *in vitro* grown *M. bovis* and/or lab adapted strains (AN5, BCG). An alternative approach would be to compare field strains of *M. bovis* to nontuberculous mycobacteria (NTM) to which cattle and captive wildlife are exposed using genomic/proteomic approaches. Identifying the NTM to use in these comparisons is critical to this approach. Early work was performed to identify mycobacteria found in the agricultural environments (e.g. soil, water, feed, other farm animals) [17-20]. A second approach has been to identify mycobacteria cultured from skin/γ-interferon test reactors (reviewed by [21]). These approaches have resulted in the identification of a number of potential candidates; however, a more systematic approach may be needed to identify mycobacterial infection/exposure in the United States. Regional differences may also play a role in diagnostic failure because mycobacterial exposure in the deserts of the Southwest may be fundamentally different than those of the Northern Michigan.

The United States Department of Agriculture’s National Veterinary Services Laboratories (NVSL) is the primary *M. bovis* diagnostic laboratory in the Untied States and has the most comprehensive database of mycobacterial isolates from animals in the United States. Using this database a retrospective study performed to identify mycobacteria isolated from tissues submitted for clinical evaluation that may interfere with current diagnostic tests.

## Results

Mycobacteria were isolated from 2,588 of 37,841 animals or pooled samples submitted to the NVSL between 1 January 2004 and 9 August 2011. These samples were collected in the United States from naturally infected animals. The majority of the samples were from domestic cattle (28,846) and cervids (4,471) with canidae (929), suidae (599), aves (510), felidae (476), bison (453), non-human primates (416), procyonidae (261), cetacea (155), elephants (146), and exotic ruminants (94) contributing the majority of the remaining samples. The remaining samples were from disparate species such as fish, sharks, camelids, hyrax, frogs, tortoise, horses, and other species.

No mycobacteria were isolated from 92% (34,867 of 37,841) of samples submitted. Fifty-five known mycobacterial species/complexes were isolated during this time period from all animal species (Additional file 1: Tables S1 and S2). The most frequently isolated mycobacteria were members of the *M. avium* complex. In general *M. avium* species were not further characterized unless they were suspected to be *M. avium* ssp. *paratuberculosis* (MAP). Samples specifically submitted to NVSL for MAP diagnostics were excluded from this study. The second species most commonly isolated was *M. bovis* followed by *M. fortuitum*. Mycobacteria that were unable to be speciated comprised the fourth group. This group represents mycobacterial species that could not be confidently identified to the species level.

### Mycobacteria isolated from cattle

NVSL received samples from 28,846 domestic cattle during the study period. Mycobacteria were isolated from 1,439 (5%). *M. bovis* comprised the majority (460, 32.0%) of the isolates. *M. avium complex* species were isolated from 367 (25.5%). *M. fortuitum*/*M. fortuitum* complex (145) made up 10.1% of the isolates recovered from cattle. Mycobacteria that could not be speciated accounted for 6.9% (100). *M. smegmatis* was isolated from 3.7% of the samples. The remaining non-*M. bovis* mycobacteria were isolated from 2% or less of the animals (Table 1).

**Table 1:** Mycobacteria isolated from domestic cattle

		**Abattoir**	**Field**
**Isolate**	**Total**	**Surveillance**	**Samples**
No. Submissions	28738	2215	6588
Mycobacterium bovis	460	203	257
Mycobacterium avium complex	367	250	117
Mycobacterium fortuitum	119	54	65
Unable to Speciate	100	49	51
Mycobacterium smegmatis	53	7	46
Mycobacterium kansasii	28	13	15
Mycobacterium fortuitum complex	26	7	19
Mycobacterium terrae complex	25	13	12
Mycobacterium pulveris	17	2	15
Mycobacterium intermedium	17	5	12
Mycobacterium porcinum	14	10	4
Mycobacterium nonchromogenicum	14	8	6
Mycobacterium simiae	13	5	8
Mycobacterium peregrinum	9	8	1
Mycobacterium shimoidei	8	6	2
Mycobacterium terrae	8	3	5
Mycobacterium thermoresistibile	7	2	5
Mycobacterium lentiflavum	5	1	4
Mycobacterium interjectum	4	3	1
Mycobacterium vaccae	4		4
Mycobacterium asiaticum	4	3	1
Mycobacterium neoaurum	3	1	2
Mycobacterium chelonae complex	3		3
Mycobacterium flavescens	3	3	
Mycobacterium monacense	2	1	1
Mycobacterium scrofulaceum	2	2	
Mycobacterium phlei	2		2
Mycobacterium species	2		2
Mycobacterium szulgai	2	2	
Mycobacterium wolinskyi	1		1
Mycobacterium triplex	1		1
Mycobacterium septicum	1		1
Mycobacterium gadium	1		1
Mycobacterium goodii	1		1
Mycobacterium triviale	1		1
Mycobacterium palustre	1		1
Mycobacterium engbackii	1	1	
Mycobacterium duvalii	1	1	
Mycobacterium abscessus	1		1

#### Mycobacteria isolated from abattoir and field surveillance

The primary goal of this study was to identify mycobacteria that may be interfering with current diagnostic tests. Samples submitted to NVSL are primarily submitted through two mechanisms: (1) abattoir surveillance; or (2) field collected tissue samples. In the United States, federal and state animal health inspectors examine carcasses at the abattoir for the presence of a number of diseases including tuberculosis. When gross lesions are identified that are compatible with tuberculosis, tissues are collected and sent to NVSL for diagnosis [22]. Mycobacteria isolated from these lesions will allow identification of mycobacteria that may produce pathology similar to *M. bovis*; however the test status of these animals is not known. Additional laboratory work would be needed to determine if these mycobacteria interfere with the diagnostic tests. The reason for each field sample submission is often not known nor routinely recorded at the laboratory; however, based on broad summary data from the laboratory, most field samples submitted were from test positive cattle. Unlike abattoir surveillance where visible granulomas trigger sample collection; non-lesioned tissues are routinely submitted for mycobacterial culture from field collected tissue samples. Examination of the mycobacteria isolated from field-collected samples may provide the most promising data since these samples come from test positive animals. Examining these two mechanisms independently may provide insight into the effect of environmental mycobacteria on diagnostics. We hypothesize that NTM recovered from field-collected samples are more likely to interfere with current antemortem diagnostic tests.

Mycobacteria were more likely to be isolated from field samples than abattoir submissions (p < 0.0001). Mycobacteria were isolated from 729 of 22,246 animals (3.3%) from abattoir surveillance compared to 710 of 3,360 (10.7%) animals from field surveillance. The top isolates from field surveillance cases in descending order were; *M. bovis* (36.2%), *M. avium* complex (16.5%), *M. fortuitum*/*fortuitum complex* (11.8%), mycobacteria that could not be speciated (7.2%), *M. smegmatis* (6.5%)*, M. kansasii* (2.1%), and *Mycobacterium pulveris* (2.1%) (Table 1). The remaining isolates comprised less than 2% of the species isolated.

Mycobacteria isolated from abattoir surveillance were: *M. avium* complex (34.3%), *M. bovis* (27.8%), *M. fortuitum/fortuitum* complex (8.4%), mycobacteria that could not be speciated (6.7%), *M. kansasii* (1.8%), *M. terrae* complex (1.8%) and *M. porcinum* (1.4%). The remaining isolates comprised <1% of the mycobacterial species isolated from abattoir surveillance cases (Table 1).

#### Mycobacteria isolated from tissues with mycobacterium bovis compatible lesions

Histopathological examination was performed on field samples with gross lesions. Mycobacteria other than *M. bovis* were cultured from 11 field samples that contained microscopic lesions compatible with *M. bovis* infection but from which no *M. bovis* was cultured. The NTM isolated from these samples were: *M. avium* complex (5), unable to speciate (2), *M. smegmatis* (1), *M. kansasii* (1), and *M. lentiflavum* (1). NTM were cultured from 22 samples submitted from abattoir surveillance that contained lesions that were histopathologically compatible with *M. bovis* infection. These were primarily members of the *M. avium* complex (18). *M. intermedium* and *M. kansasii* were cultured from two abattoir submissions each.

#### Seasonal isolation of mycobacteria

Samples were submitted to NVSL year around. There was no apparent difference in the mycobacterial species isolated at different times of the year or by submission type. *M. bovis* was isolated at a similar rate regardless of the time of the year or type of submission (data not shown).

#### Isolation of mycobacteria from different regions in the United States

Anecdotal data from interferon-γ release assays and serum based diagnostic test validation has suggested that cross-reactivity to NTM may be different between regions of the United States. Isolates were tabulated by the region of the United States from which they originated and by submission type. Overall, *M. avium* complex was most frequently isolated from abattoir surveillance whereas *M. bovis* was the predominate isolate from field surveillance cases.

Mycobacteria isolated from samples submitted for abattoir surveillance primarily resulted in isolation of *M. avium* complex followed by *M. bovis*, *M. fortuitum*, uncharacterized mycobacteria, and *M. kansasii*. Isolations from abattoir surveillance from the South West region were predominantly *M. bovis* followed by *M. avium* complex. In the Mid-Atlantic and Southeast regions uncharacterized species played a more significant role (Table 2).

**Table 2:** Regional differences in mycobacteria isolated from samples collected from cattle as part of the abattoir surveillance program

		**Pacific**			**South**	**Central**	**Great**	**North**	**Mid**	**South**	
**Isolate**	**Total**	**North**	**Pacific**	**Mountain**	**West**	**Plains**	**Lakes**	**North**	**Atlantic**	**East**	**Unknown**
No. Submissions	22813	313	203	2577	4427	4031	3059	420	1261	1255	5267
No. Mycobactria Isolated	663	9	3	55	258	82	50	5	55	32	114
Mycobacterium avium complex	250	6	2	24	54	35	24	2	18	14	71
Mycobacterium bovis	203	1	1	15	144	21	12		3	1	5
Mycobacterium fortuitum	54			4	23	9	2		5	3	8
Unable to Speciate	49			3	14	3	3	1	7	8	10
Mycobacterium kansasii	13	1			2	3	1	1	2		3
Mycobacterium terrae complex	13			2	3	3	1			1	3
Mycobacterium porcinum	10								9		1
Mycobacterium peregrinum	8							1	7		
Mycobacterium nonchromogenicum	8	1		1		1	1		1	1	2
Mycobacterium fortuitum complex	7			1	1		1		1		3
Mycobacterium smegmatis	7			1	1				1	2	2
Mycobacterium shimoidei	6			3	1		1			1	
Mycobacterium intermedium	5					2	1				2
Mycobacterium simiae	5			1	3				1		
Mycobacterium asiaticum	3				2						1
Mycobacterium terrae	3				1	1	1				
Mycobacterium flavescens	3				1	1					1
Mycobacterium interjectum	3				3						
Mycobacterium szulgai	2				1					1	
Mycobacterium thermoresistibile	2					1	1				
Mycobacterium pulveris	2					1	1				
Mycobacterium scrofulaceum	2				1						1
Mycobacterium lentiflavum	1				1						
Mycobacterium neoaurum	1				1						
Mycobacterium engbackii	1										1
Mycobacterium monacense	1				1						
Mycobacterium duvalii	1					1					

Isolation of mycobacteria from field surveillance submissions resulted in a more complex pattern. In general, *M. bovis* was most frequently isolated. There were several notable exceptions. In the Pacific region more *M. avium* complex and uncharacterized mycobacteria were isolated than *M. bovis*. In addition, more *M. avium* complex and *M. smegmatis* were isolated than *M. bovis* in the Southeast region. There were two few samples to make definitive conclusions from the Pacific North, Northeast, and Mid-Atlantic regions (Table 3).

**Table 3:** Regional differences in mycobacteria isolated from field collected samples from cattle

		**Pacific**			**South**	**Central**	**Great**	**North**	**Mid**	**South**	
**Isolate**	**Total**	**North**	**Pacific**	**Mountain**	**West**	**Plains**	**Lakes**	**North**	**Atlantic**	**East**	**Unknown**
No. Submissions	6642	14	2484	402	1230	324	1825	23	15	199	126
No. Mycobactria Isolated	722	2	207	82	109	30	252	1	3	18	18
Mycobacterium bovis	257		20	44	65	3	122			2	1
Mycobacterium avium complex	117	2	46		8	9	36	1	1	6	8
Mycobacterium fortuitum	65		25	29	2	1	5			1	2
Unable to Speciate	51		26	2	6	2	13			1	1
Mycobacterium smegmatis	46		6	1	1	4	26		2	3	3
Mycobacterium fortuitum complex	19		7		3	3	4				2
Mycobacterium pulveris	15		10		1	3	1				
Mycobacterium kansasii	15		13		1	1					
Mycobacterium intermedium	12		8			2	1			1	
Mycobacterium terrae complex	12		1		1		9			1	
Mycobacterium simiae	8		5		1		2				
Mycobacterium nonchromogenicum	6		1	2			2			1	
Mycobacterium terrae	5		1	1	2		1				
Mycobacterium thermoresistibile	5			1	2		2				
Mycobacterium lentiflavum	4						4				
Mycobacterium vaccae	4						4				
Mycobacterium porcinum	4		3		1						
Mycobacterium chelonae complex	3		1				1				1
Mycobacterium phlei	2		1				1				
Mycobacterium shimoidei	2		1		1						
Mycobacterium neoaurum	2						1			1	
Mycobacterium species	2				1		1				
Mycobacterium goodii	1						1				
Mycobacterium monacense	1			1							
Mycobacterium asiaticum	1									1	
Mycobacterium palustre	1						1				
Mycobacterium wolinskyi	1		1								
Mycobacterium interjectum	1						1				
Mycobacterium triviale	1						1				
Mycobacterium triplex	1				1						
Mycobacterium gadium	1		1								
Mycobacterium peregrinum	1						1				
Mycobacterium abscessus	1		1								
Mycobacterium septicum	1						1				

#### Isolation of multiple mycobacteria from the same animal

Each submitted sample was cultured on at least one liquid medium and three solid media. Culture on solid media provided the opportunity to potentially identify co-infections. There were 13 confirmed instances where two mycobacterial species were isolated from the same animal. *M. bovis* was isolated with *M. smegmatis* (n = 4), *M. lentiflavum* (n = 1), and *M. avium* complex (n = 1). *M. neoaurum* was isolated with *M. fortuitum* (n = 1) and *M. smegmatis* (n = 1). The remaining co-isolates were: *M. simiae* and *M. wolinskyi*; *M. smegmatis* and unable to speciate; *M. terrae* and *M. thermoresistible*; *M. asiaticum* and *M. nonchromogenicum*; *M. fortuitum* and *M. pulveris*. This not an exhaustive list since NTM were generally not further identified when *M. bovis* had already been isolated from the animal. Most of these were recovered from the same tissue but from different media types, such as 7H11 agar and L-J. Occasionally culturing multiple tissues from the same animal would recover more than one mycobacteria. These data suggest that animals can be co-infected with at least two mycobacteria at the same time.

### Mycobacteria isolated from cervids

NVSL received samples from 4,471 cervids in the United States between 1 January 2004 and 9 October 2011. Samples were submitted from both farmed and free-ranging cervids. Submission usually consisted of lymph nodes of the head, especially the retropharyngeal lymph node, and lesions from the lung, when present. Samples submitted from farmed cervids were cultured individually. Submissions from free-ranging cervids were tested individually if lesioned, and in areas of active surveillance such as around positive cattle herds. If deer were non-lesioned and outside of an active surveillance zone retropharyngeal lymph nodes from up to 5 deer were pooled. Pooled samples were treated as an individual for the purposes of this study. Mycobacteria were isolated from 482 of the 4,471 samples submitted. *M. bovis* (119, 24.7%) was the most commonly isolated mycobacterium, followed by *M. avium* complex (70, 14.5%). Uncharacterized mycobacteria comprised the third group (34. 7.1%). The next two were *M. terrae*/*terrae complex* (25, 5.2%) and *M. fortuitum/fortuitum* complex (20, 4.1%) (Table 4). The remaining isolates were isolated 10 or fewer times during this time period.

**Table 4:** Mycobacteria isolated from cervids

**Isolate**	**Count**
No. Submissions	4311
Mycobacterium bovis	119
Mycobacterium avium complex	70
Unable to Speciate	34
Mycobacterium terrae complex	14
Mycobacterium terrae	11
Mycobacterium fortuitum	11
Mycobacterium fortuitum complex	9
Mycobacterium kansasii	8
Mycobacterium abscessus	8
Mycobacterium nonchromogenicum	7
Mycobacterium smegmatis	6
Mycobacterium simiae	3
Mycobacterium asiaticum	2
Mycobacterium intermedium	2
Mycobacterium paratuberculosis	2
Mycobacterium septicum	2
Mycobacterium nebraskense	1
Mycobacterium pulveris	1
Mycobacterium triviale	1
Mycobacterium holsaticum	1
Mycobacterium shimoidei	1
Mycobacterium thermoresistibile	1
Mycobacterium gastri	1
Mycobacterium scrofulaceum	1
Mycobacterium engbackii	1
Mycobacterium kubicae	1
Mycobacterium goodii	1
Mycobacterium gadium	1
Mycobacterium szulgai	1
Mycobacterium alvei	1

Samples from axis deer, caribou, fallow deer, mule deer, red deer, sika deer, white-tailed deer, elk, moose, muntjac and reindeer were submitted to NVSL for diagnosis (Table 5). Mycobacteria isolated from individual host species was similar where significant numbers of samples were submitted. Only 27 samples were submitted from abattoir surveillance. Of these, *M. bovis* was isolated from 6 of the samples (5 from red deer and 1 from an elk), 3 *M. kansasii* (elk) and 1 *M. avium* complex (elk). Mycobacteria were not isolated from the remainder of the submissions from abattoir surveillance.

**Table 5:** Mycobacteria isolated from field collected samples from cervids

**Cervid**	**Isolate**	**Count**
Antelope	Mycobacterium avium complex	1
Deer^a^	No. Submissions	3693
	Mycobacterium bovis	35
	Mycobacterium avium complex	29
	Unable to Speciate	20
	Mycobacterium terrae complex	14
	Mycobacterium terrae	11
	Mycobacterium fortuitum	10
	Mycobacterium abscessus	7
	Mycobacterium fortuitum complex	6
	Mycobacterium smegmatis	5
	Mycobacterium asiaticum	2
	Mycobacterium nonchromogenicum	2
	Mycobacterium shimoidei	1
	Mycobacterium gastri	1
	Mycobacterium alvei	1
	Mycobacterium kubicae	1
	Mycobacterium engbackii	1
	Mycobacterium kansasii	1
	Mycobacterium septicum	1
	Mycobacterium triviale	1
	Mycobacterium scrofulaceum	1
	Mycobacterium pulveris	1
Elk^b^	No. Submissions	390
	Mycobacterium bovis	46
	Mycobacterium avium complex	32
	Unable to Speciate	13
	Mycobacterium nonchromogenicum	5
	Mycobacterium intermedium	2
	Mycobacterium kansasii	2
	Mycobacterium simiae	1
	Mycobacterium septicum	1
	Mycobacterium abscessus	1
	Mycobacterium goodii	1
	Mycobacterium fortuitum	1
	Mycobacterium szulgai	1
	Mycobacterium gadium	1
	Mycobacterium fortuitum complex	1
	Mycobacterium thermoresistibile	1
Fallow Deer	No. Submisions	77
	Mycobacterium bovis	32
	Mycobacterium avium complex	8
	Mycobacterium fortuitum complex	2
	Mycobacterium simiae	2
	Mycobacterium smegmatis	1
	Mycobacterium nebraskense	1
	Mycobacterium holsaticum	1
	Unable to Speciate	1

^a^ Includes white-tailed and mule deer.

^b^ Includes Red and Sika Deer.

The majority of cervid samples submitted were from field surveillance. Most of the isolates (49.2%) from deer were not characterized beyond confirmation they were not *M. bovis*. *M. bovis* was isolated from 11.8% of the animals sampled, followed by *M. avium* complex (10.0%), *M. terrae/terrae* complex (8.4%), uncharacterized mycobacteria (6.8%), and *M. fortuitum/fortuitum* complex (5.4%) (Table 5). Mycobacteria that were characterized in Elk (including red deer) were *M. bovis* (33.0%), *M. avium* complex (23.5%) and *M. nonchromogenicum* (3.0%) (Table 5).

#### Mycobacteria isolated from tissues with mycobacterium bovis compatible lesions

Lesions that were compatible with tuberculosis were identified histologically in cervids; however, *M. bovis* was the only mycobacterium that was isolated from these tissues.

#### Seasonal isolation of mycobacteria from cervids

Cervid samples were primarily submitted during the fall (27%) and winter months (41%). The remaining samples were submitted during the spring (16%) and summer (16%) months. All of the summer samples were received over the course of two years. This seasonal submission is expected since survey of hunter killed deer plays a substantial role in field surveillance of cervids in the United States. There was no evidence that isolation of mycobacteria from tissues was seasonal (Table 6).

**Table 6:** Seasonal differences in mycobacteria isolated from deer

**Isolate**	**Total**	**Winter**^**a**^	**Spring**^**b**^	**Summer**^**c**^	**Fall**^**d**^
No. Submissions	3697	1565	550	588	994
Mycobacterium bovis	35	11	13	2	9
Mycobacterium avium complex	29	13	3	3	10
Unable to Speciate	20	10	5	3	2
Mycobacterium terrae complex	14	1		6	7
Mycobacterium terrae	11			8	3
Mycobacterium fortuitum	10	2	2	5	1
Mycobacterium abscessus	7	1		3	3
Mycobacterium fortuitum complex	6	2	1	1	2
Mycobacterium smegmatis	5	1	1		3
Mycobacterium nonchromogenicum	2	2			
Mycobacterium asiaticum	2				2
Mycobacterium scrofulaceum	1				1
Mycobacterium triviale	1			1	
Mycobacterium kubicae	1	1			
Mycobacterium kansasii	1	1			
Mycobacterium alvei	1				1
Mycobacterium gastri	1				1
Mycobacterium shimoidei	1		1		
Mycobacterium engbackii	1			1	
Mycobacterium pulveris	1	1			
Mycobacterium septicum	1				1

^a^December, January, February ^b^March, April, May ^c^June, July, August ^d^September, October, November.

#### Isolation of multiple mycobacteria from the same animal

Multiple mycobacteria were isolated from the same animal in 7 instances in cervids. Three mycobacteria, *M. fortuitum complex, M. holsaticum,* and *smegmatis* were isolated from one fallow deer. Other dual isolates from fallow deer included: *M. nebraskense* and *M. simiae*; and *M. avium* complex with *M. simiae*. Multiple isolations from elk included *M. bovis* with *M. thermoresistible* (n = 1) and *M. nonchromogenicum* (n = 1). *M. fortuitum* and *M. smegmatis* were isolated from a white-tailed deer.

## Discussion

Sequencing the genomes of the environmental mycobacteria that are encountered by livestock may provide researchers the ability to identify bacterial proteins or nucleic acid targets that will not cross react with *M. bovis*; thus enabling the development of assays with increased specificity and sensitivity. Identifying the mycobacteria that infect livestock and wildlife may enable the rational prioritization of the mycobacterial genomes to sequence to aid diagnostic test development.

A number of mycobacteria reported here have been isolated from the environment associated with cattle feed or water. Many of these have been reported to elicit reactions to PPDb based skin tests including isolates belonging to Runyan Group III [19], *M. flavescens*, *M. terrae* complex, *M. gordonae*, *M. intracellulare*[20], *M. fortuitum*, *M. smegmatis*, *M. senegalense*, *M. scrofulaceum*, (reviewed in [21]) and *M. kansasii*[23,24]. In addition to cattle, *M. fortuitum* has been reported to interfere with the skin and γ-interferon test in African Buffalo [13,14]. Immunological cross-reaction between these mycobacteria and PPDa and PPDb suggest that immunologically similar proteins exist between mycobacteria. Cross-reaction reduces specificity and sensitivity of diagnostic tests.

*M. bovis* and *M. avium* complex species are the most common mycobacteria isolated from cervids in the United States. These results are consistent with those reported for New Zealand [25]. In contrast to cattle, more *M. terrae/terrae* complex were isolated from cervids than *M. fortuitum/fortuitum* complex.

Cervids may be more susceptible to mycobacterial infections. In this study, mycobacteria were isolated at a rate of 107 per thousand cases compared to cattle with a rate of 49.9 per thousand cases. If a herd is infected with a greater range of environmental mycobacteria there is a greater probability that the immune response to the environmental mycobacteria may cross-react with PPDa and/or PPDb thus reducing the specificity and/or sensitivity of the diagnostic test.

There are two caveats to the current data. First, not all isolates were identified to the species level. These totals may be different if all samples were speciated as far as possible. This may be most prominent in the *M. avium* complex isolates. Hughes et al. report that when improve molecular techniques were applied members of the *M. avium* group resulted in identification of a larger number of mycobacterial species [26]. We reason that the major isolates would be similar, but some of the minor species may play a larger role than currently indicated by the data. Not all isolates were speciated due to the priority of the diagnostic laboratory to identify and report *M. bovis* infections. Some isolates were not speciated because they could not be confidently assigned to the species level (Unable to Speciate). This inability to speciate each mycobacterial isolate was also reported by Hues et al. [26] in Northern Ireland, suggesting that not all mycobacterial species have been identified.

A second caveat is that not all mycobacteria are cultivatable. The possibility exists that the mycobacteria that cause significant interference with current diagnostic tests are unculturable. A prime example of this scenario is the infrequent yet persistent finding of CCT positive cattle with lesions only found in the skin. Histopathological examination reveals the presence of acid-fast bacteria, but culture attempts are always unsuccessful.

With the advent of next generation sequencing, the possibility exists that mycobacterial species could be sequenced with little capital investment. Regions or proteins unique to *M. bovis* could be derived by comparing the *M. bovis* genome with environmental mycobacteria. These unique regions could then be tested for use in protein or nucleic acid based tests. The problem with this approach has been the difficulty in selecting the environmental mycobacteria to sequence. Here we report the mycobacteria isolated from cattle and cervids in the United States. These data provide a rational for selection of mycobacteria for genome sequencing to aid in the development of improved diagnostic tests.

## Conclusions

Between 1 January 2004 and 9 October 2001, 2,389 nontuberculous mycobacteria were isolated from samples submitted for clinical diagnosis of *M. bovis*. In cattle, *M. avium* complex, *M. fortuitum/fortuitum* complex, *M. smegmatis,* and *M. kansasii* were the predominate species. Un-speciated Mycobacteria were the most commonly isolated category of mycobacteria isolated from deer followed by *M. bovis*, *M. avium* complex, *M. terrae/terrae* complex then *M. fortuitum/fortuitum* complex.

## Methods

### Mycobacterial culture and identification

The samples submitted from cattle and cervids are collected under the purview of the national eradication program using trained state and federal veterinary medical officers. This collection methodology results in high quality sample submission. Samples were shipped in sodium borate as described previously [27,28]. Tissue samples were trimmed of fat and some connective tissue, soaked in a 0.065% solution of sodium hypochlorite for 15 ± 5 minutes, placed in individual sterilized pint jars, covered with phenol red nutrient broth and then homogenized. Samples were transferred to 50 ml centrifuge tubes and decontaminated with a 0.8-1.8% final concentration of sodium hydroxide for 7–10 minutes and neutralized to effect with hydrochloric acid. Samples were then centrifuged at 4,800 × g for 20 minutes. Media selection depended upon the species of the samples, suspected disease and risk of infection. In general at least one liquid media (either BACTEC 12B (Becton Dickinson, Sparks, MD, USA) and MGIT 960 (Becton Dickinson, Sparks, MD, USA) were used and a minimum of 3 solid media types were used for each sample. Solid media used from most common to least common were: 7H11 supplemented with calf serum, lysed sheep blood, sodium pyruvate, and malachite green; 7H10 prepared with pyruvate; Mycobactosel LJ (Becton Dickinson, Sparks, MD, USA); Lowenstein Jensen (Becton Dickinson, Sparks, MD, USA); Stonebrinks; 7H11 supplemented with calf serum, lysed sheep blood, glycerol, and malachite green; 7H10 prepared with glycerol; and Herrold’s Egg Yolk Agar. In general we did not use mycobactin J supplemented media unless specifically asked by the submitter. Detailed performance of this decontamination method and media is published elsewhere [29]. Commercial DNA probes (Gen-Probe, San Diego, CA) were used to identify *M. tuberculosis* complex (MTBC) and *M. avium* complex organisms. MTBC probe positive isolates were subject to spoligotyping and niacin/nitrate biochemical testing to speciate *M. bovis*[30,31]. Prior to 2009, isolates submitted for *M. bovis* culture that were DNA probe positive for M. avium complex were not tested further. NTM identification was based from partial 16S rDNA sequencing and biochemical testing from 2004 through 2009 [31,32]. Sequences were compared using the RIDOM database [33]. An isolate with a nucleotide match greater than 99.3% with no biochemical mismatches were identified to the species level. Isolates with nucleotide homology greater than 99.3% with biochemical mismatches were reported at the complex level (e.g. Mycobacterium avium complex). When the nucleotide match was greater than 98.5% but less than 99.3% and it fell with a complex the isolate was reported at the complex level. Isolates that had nucleotide homology was less than 98.5% but greater than 96% were reported as “unable to speciate”. After 2009, partial sequencing of *rpoB* and *hsp65* regions replaced biochemical testing when needed for higher resolution [34,35]. At this time, DNA probes for M. avium complex were abandoned and *rpoB* sequencing was used in place of this procedure. This provided greater species resolution and those results are published elsewhere [36]. Those same isolates are included in this paper, but only at the complex level to maintain consistency to prior years.

### Data collection and database development

The following information was obtained from the NVSL databases; accession number, date sample received at NVSL, submission type (abattoir or field), result of mycobacterial identification, species or source of sample, histological result, and the state of origin. MySQL (version 5.5.12) was used to build a custom database for data analysis. Initial analysis included filtering to remove redundant data (i.e. when *M. bovis* was isolated from more than one tissue the duplicates were removed so that each animal only appears once in the data base). The only time an animal appears more than once is when more than one mycobacterium was isolated from that animal.

### Designation of season and region

Data were aggregated by the time of year the sample was received for some analysis. The following criteria were used: Winter months were December through February; Spring months were March through May; Summer months were June through August; and Fall months were September through November. When data were aggregated by Region of the United States the following criteria were used: Central Plains included Iowa, Illinois, Kansas, Missouri, Nebraska; Great Lakes included Indiana, Michigan, Minnesota, Ohio, Wisconsin; Mid-Atlantic included District of Columbia, Maryland, New Jersey, Pennsylvania, Virginia, West Virginia; Mountain included Colorado, Idaho, Montana, North Dakota, South Dakota, Utah, Wyoming; Northeast included Connecticut, Delaware, Massachusetts, New Hampshire, New York, Rhode Island, Vermont, Maine; Pacific encompassed California, Hawaii, Nevada; Pacific North included Alaska, Oregon, Washington; Southeast encompassed Alabama, Arkansas, Florida, Georgia, Kentucky, Louisiana, Mississippi, North Carolina, South Carolina, Tennessee; and the Southwest included Arizona, New Mexico and Texas.

### Statistical analysis

Chi-square test was used to test for the association between isolation of mycobacteria and type of submission (field vs abattoir cases) in a 2×2 contingency table. The test was applied using Prism version 5.0d (GraphPad Software Inc., San Diego, CA).

## Competing interests

The authors declare that they have no competing interests.

## Authors’ contributions

TCT, SRA, and BH conceived the study and participated in developing quality control parameters of the data. TCT built the database and analyzed the data. All authors participated in data interpretation. Manuscript was written by TCT, reviewed and edited by SRA, BH, MVP, WRW. All authors read and approved the final manuscript.

## Supplementary Material

Additional file 1: Table S1 Mycobacterial Species Isolated between 1 Jan 2004 and 9 Aug 2011. **Table S2.** Summary of mycobacteria isolated from tissues collected from species other than domestic cattle and deer from the United State between 1 Jan 2004 and 9 Aug 2011. Click here for file
