# Supplementary material for: Isolation of mycobacteria from clinical samples collected in the United States from 2004 to 2011
Source: BMC Vet Res. 2013 May 8;9:100. doi: 10.1186/1746-6148-9-100 (PMC3654946; doi:10.1186/1746-6148-9-100)
Supplement: Additional file 1: Table S1 — Mycobacterial Species Isolated between 1 Jan 2004 and 9 Aug 2011. Table S2. Summary of mycobacteria isolated from tissues collected from species other than domestic cattle and deer from the United State between 1 Jan 2004 and 9 Aug 2011. [file 1746-6148-9-100-S1.pdf]

Supplemental tables

Table 1: Mycobacterial Species Isolated between 1 Jan 2004 and 9 Aug 2011.

Species	Count
Mycobacterium avium complex	832
Mycobacterium bovis	644
Mycobacterium fortuitum	170
Mycobacterium smegmatis	92
Mycobacterium fortuitum complex	56
Mycobacterium kansasii	55
Mycobacterium terrae complex	43
Mycobacterium tuberculosis	42
Mycobacterium paratuberculosis	35
Mycobacterium terrae	31
Mycobacterium abscessus	25
Mycobacterium nonchromogenicum	23
Mycobacterium chelonae complex	22
Mycobacterium pulveris	22
Mycobacterium intermedium	19
Mycobacterium marinum	18
Mycobacterium asiaticum	18
Mycobacterium simiae	18
Mycobacterium chelonae	17
Mycobacterium porcinum	14
Mycobacterium thermoresistibile	11
Mycobacterium peregrinum	11
Mycobacterium szulgai	10
Mycobacterium shimoidei	10
Mycobacterium lentiflavum	7
Mycobacterium neoaurum	6
Mycobacterium interjectum	5
Mycobacterium vaccae	5
Mycobacterium goodii	4
Mycobacterium septicum	4
Mycobacterium scrofulaceum	3
Mycobacterium phlei	3
Mycobacterium flavescens	3
Mycobacterium monacense	3
Mycobacterium gordonae	3
Mycobacterium haemophilum	3
Mycobacterium engbackii	2
Mycobacterium ulcerans	2
Mycobacterium gadium	2
Mycobacterium triviale	2
Mycobacterium genavense	1
Mycobacterium triplex	1
Mycobacterium duvalii	1
Mycobacterium wolinskyi	1

Continued...

Species	Count
Mycobacterium paraffinicum	1
Mycobacterium gilvum	1
Mycobacterium smegmatis complex	1
Mycobacterium kubicae	1
Mycobacterium holsaticum	1
Mycobacterium palustre	1
Mycobacterium alvei	1
Mycobacterium nebraskense	1
Mycobacterium marseillense	1
Mycobacterium bolletii	1
Mycobacterium gastri	1

Table 2: Summary of mycobacteria isolated from tissues collected from species other than domestic cattle and deer from the United State between 1 Jan 2004 and 9 Aug 2011.

Sample Origin	Mycobacterium	Count
Amphibia	Mycobacterium marinum	9
	Mycobacterium species[a]	3
	Mycobacterium chelonae	2
	Mycobacterium ulcerans	1
	Mycobacterium avium complex	1
	Mycobacterium terrae	1
	Mycobacterium fortuitum	1
	Mycobacterium fortuitum complex	1
	Mycobacterium species	1
Aves	Mycobacterium avium complex	69
	Mycobacterium species	10
	Mycobacterium tuberculosis	1
	Mycobacterium fortuitum complex	1
	Mycobacterium tuberculosis complex	1
	Mycobacterium genavense	1
	Mycobacterium pulveris	1
	Mycobacterium fortuitum	1
	Mycobacterium kansasii	1
Bison	Mycobacterium species	10
	Mycobacterium avium complex	8
	Mycobacterium pulveris	3
	Mycobacterium smegmatis	1
	Mycobacterium neoaurum	1
Camelidae	Mycobacterium avium complex	2
	Mycobacterium paratuberculosis	1
Canidae	Mycobacterium avium complex	50
	Mycobacterium bovis	38
	Mycobacterium species	21
	Mycobacterium abscessus	7
	Mycobacterium smegmatis	3
	Mycobacterium fortuitum	3
	Mycobacterium kansasii	1
	Mycobacterium interjectum	1
	Mycobacterium bolletii	1
Caprinae	Mycobacterium avium complex	6
	Mycobacterium kansasii	3
	Mycobacterium paratuberculosis	3
	Mycobacterium species	1
Cetacea	Mycobacterium species	14

Continued...

Sample Origin	Mycobacterium	Count
	Mycobacterium bovis	8
	Mycobacterium tuberculosis complex	2
	Mycobacterium avium complex	2
	Mycobacterium neoaurum	1
	Mycobacterium chelonae	1
	Mycobacterium fortuitum complex	1
Elephantidae	Mycobacterium species	46
	Mycobacterium avium complex	27
	Mycobacterium tuberculosis	14
	Mycobacterium terrae	6
	Mycobacterium szulgai	3
	Mycobacterium fortuitum complex	2
	Mycobacterium smegmatis	2
	Mycobacterium septicum	1
	Mycobacterium nonchromogenicum	1
	Mycobacterium goodii	1
	Mycobacterium monacense	1
	Mycobacterium paraffinicum	1
	Mycobacterium tuberculosis complex	1
	Mycobacterium fortuitum	1
Equidae	Mycobacterium avium complex	2
	Mycobacterium species	1
Erinaceidae	Mycobacterium haemophilum	1
Exotic Ruminants[b]	Mycobacterium avium complex	8
	Mycobacterium kansasii	5
	Mycobacterium simiae	1
	Mycobacterium species	1
	Mycobacterium fortuitum complex	1
Felidae	Mycobacterium species	43
	Mycobacterium avium complex	27
	Mycobacterium smegmatis	26
	Mycobacterium fortuitum	21
	Mycobacterium fortuitum complex	8
	Mycobacterium thermoresistibile	3
	Mycobacterium chelonae complex	3
	Mycobacterium abscessus	3
	Mycobacterium goodii	1
	Mycobacterium lentiflavum	1
	Mycobacterium smegmatis complex	1
Fish	Mycobacterium marinum	6
	Mycobacterium species	5
	Mycobacterium haemophilum	2
	Mycobacterium lentiflavum	1

Continued...

Sample Origin	Mycobacterium	Count
	Mycobacterium ulcerans	1
	Mycobacterium chelonae complex	1
	Mycobacterium chelonae	1
	Mycobacterium abscessus	1
Giraffidae	Mycobacterium avium complex	1
	Mycobacterium kansasii	1
Leporidae	Mycobacterium avium complex	1
Macropodidae	Mycobacterium avium complex	10
	Mycobacterium species	1
	Mycobacterium fortuitum	1
	Mycobacterium kansasii	1
Mustelidae	Mycobacterium kansasii	2
	Mycobacterium avium complex	1
Pinnipedia	Mycobacterium fortuitum	1
	Mycobacterium species	1
Primate	Mycobacterium tuberculosis	27
	Mycobacterium avium complex	14
	Mycobacterium species	12
	Mycobacterium kansasii	5
	Mycobacterium bovis	3
	Mycobacterium szulgai	3
	Mycobacterium peregrinum	2
	Mycobacterium marseillense	1
	Mycobacterium fortuitum complex	1
	Mycobacterium simiae	1
Procyonidae	Mycobacterium species	6
	Mycobacterium bovis	6
	Mycobacterium avium complex	5
	Mycobacterium tuberculosis complex	2
	Mycobacterium terrae complex	1
	Mycobacterium smegmatis	1
Reptilia	Mycobacterium species	4
	Mycobacterium chelonae	3
	Mycobacterium chelonae complex	3
	Mycobacterium avium complex	2
	Mycobacterium abscessus	2
	Mycobacterium fortuitum	2
	Mycobacterium szulgai	1
Rhinocerotidae	Mycobacterium fortuitum complex	1

Continued…

Sample Origin	Mycobacterium	Count
Serpentes	Mycobacterium chelonae complex	5
	Mycobacterium abscessus	3
	Mycobacterium chelonae	3
	Mycobacterium gordonae	2
	Mycobacterium marinum	2
	Mycobacterium species	2
	Mycobacterium fortuitum	2
	Mycobacterium avium complex	1
	Mycobacterium fortuitum complex	1
Suidae	Mycobacterium avium complex	176
	Mycobacterium species	80
	Mycobacterium asiaticum	12
	Mycobacterium bovis	10
	Mycobacterium terrae	5
	Mycobacterium fortuitum	4
	Mycobacterium fortuitum complex	3
	Mycobacterium terrae complex	3
	Mycobacterium tuberculosis complex	2
	Mycobacterium nonchromogenicum	1
	Mycobacterium vaccae	1
	Mycobacterium neoaurum	1
Tapiridae	Mycobacterium gordonae	1
	Mycobacterium avium complex	1
Testudinidae	Mycobacterium avium complex	7
	Mycobacterium chelonae complex	3
	Mycobacterium species	3
	Mycobacterium fortuitum	2
	Mycobacterium marinum	1
	Mycobacterium gilvum	1
	Mycobacterium chelonae	1
Unkown Marine	Mycobacterium chelonae	5
	Mycobacterium chelonae complex	4
	Mycobacterium fortuitum complex	1
	Mycobacterium fortuitum	1
Unknown	Mycobacterium phlei	1
	Mycobacterium avium complex	1
Unspecified Zoo Animal	Mycobacterium avium complex	2
	Mycobacterium shimoidei	1
	Mycobacterium chelonae	1

[a] Isolate was identified as mycobacteria but were not further speciated.

[b] Ruminants other than domestic cattle and cervids listed in Table 6
